# Rearrangement moves on rooted phylogenetic networks

**DOI:** 10.1371/journal.pcbi.1005611

**Published:** 2017-08-01

**Authors:** Philippe Gambette, Leo van Iersel, Mark Jones, Manuel Lafond, Fabio Pardi, Celine Scornavacca

**Affiliations:** 1 Laboratoire d’Informatique Gaspard-Monge (LIGM), Université Paris-Est, CNRS, ENPC, ESIEE Paris, UPEM, F-77454, Marne-la-Vallée, France; 2 Delft Institute of Applied Mathematics, Delft University of Technology, Postbus 5031, 2628 CD Delft, The Netherlands; 3 Department of Mathematics and Statistics, University of Ottawa, K1N 6N5 Ottawa, Canada; 4 Laboratoire d’Informatique, de Robotique et de Microélectronique de Montpellier (LIRMM), Université de Montpellier, CNRS, 34095 Montpellier Cedex 5, France; 5 Institut de Biologie Computationnelle (IBC), 34095 Montpellier, France; 6 Institut des Sciences de l’Evolution (ISE-M), Université de Montpellier, CNRS, IRD, EPHE, 34095 Montpellier Cedex 5, France; Fred Hutchinson Cancer Research Center, UNITED STATES

## Abstract

Phylogenetic tree reconstruction is usually done by local search heuristics that explore the space of the possible tree topologies via simple rearrangements of their structure. Tree rearrangement heuristics have been used in combination with practically all optimization criteria in use, from maximum likelihood and parsimony to distance-based principles, and in a Bayesian context. Their basic components are rearrangement moves that specify all possible ways of generating alternative phylogenies from a given one, and whose fundamental property is to be able to transform, by repeated application, any phylogeny into any other phylogeny. Despite their long tradition in tree-based phylogenetics, very little research has gone into studying similar rearrangement operations for phylogenetic network—that is, phylogenies explicitly representing scenarios that include reticulate events such as hybridization, horizontal gene transfer, population admixture, and recombination. To fill this gap, we propose “horizontal” moves that ensure that every network of a certain complexity can be reached from any other network of the same complexity, and “vertical” moves that ensure reachability between networks of different complexities. When applied to phylogenetic trees, our horizontal moves—named rNNI and rSPR—reduce to the best-known moves on rooted phylogenetic trees, nearest-neighbor interchange and rooted subtree pruning and regrafting. Besides a number of reachability results—separating the contributions of horizontal and vertical moves—we prove that rNNI moves are local versions of rSPR moves, and provide bounds on the sizes of the rNNI neighborhoods. The paper focuses on the most biologically meaningful versions of phylogenetic networks, where edges are oriented and reticulation events clearly identified. Moreover, our rearrangement moves are robust to the fact that networks with higher complexity usually allow a better fit with the data. Our goal is to provide a solid basis for practical phylogenetic network reconstruction.

## Author summary

Phylogenetic networks are used to represent reticulate evolution, that is, cases in which the tree-of-life metaphor for evolution breaks down, because some of its branches have merged at one or several points in the past. This may occur, for example, when some organisms in the phylogeny are hybrids. In this paper, we deal with an elementary question for the reconstruction of phylogenetic networks: how to explore the space of all possible networks. The fundamental component for this is the set of operations that should be employed to generate alternative hypotheses for what happened in the past—which serve as basic blocks for optimization techniques such as hill-climbing. Although these approaches have a long tradition in classic tree-based phylogenetics, their application to networks that explicitly represent reticulate evolution is relatively unexplored. This paper provides the fundamental definitions and theoretical results for subsequent work in practical methods for phylogenetic network reconstruction: we subdivide networks into layers, according to a generally-accepted measure of their complexity, and provide operations that allow both to fully explore each layer, and to move across different layers. These operations constitute natural generalizations of well-known operations for the exploration of the space of phylogenetic trees, the lowest layer in the hierarchy described above.

This is a *PLOS Computational Biology* Methods paper.

## Introduction

A recent trend in evolutionary biology is the growing appreciation of reticulate evolution—which occurs when the history of a set of taxa (e.g., species, populations or genes) cannot be accurately represented as a phylogenetic tree [1, 2], because of events causing inheritance from more than one ancestor. There is a wide variety of reticulate events in nature, for example: hybrid speciation [3–5], population admixture [6–8] horizontal gene transfer [9–11] and genomic recombination [12–14]. These phenomena are often of interest to different communities of researchers (e.g., in plant biology, population genetics, microbiology, epidemiology), meaning that different approaches and terminologies are in use in these fields.

**Fig 1:**
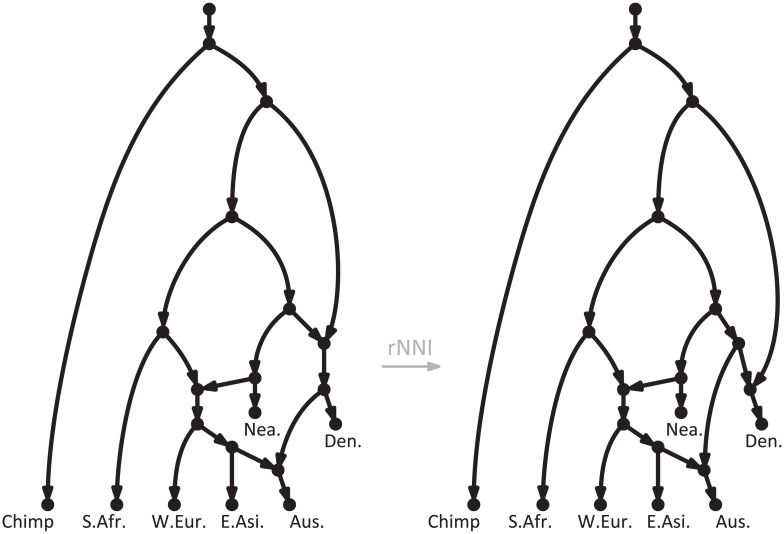
Phylogenetic network showing hypothetical evolutionary scenarios relating modern human populations and their closest relatives. On the left, a slightly simplified version of an admixture graph from a recent publication on human diversity [8]. On the right, an alternative scenario obtained by applying one of the rearrangement moves that we define here (an rNNI), which essentially swaps the order of the two events immediately ancestral to the Denisovans. S.Afr.: Sub-Saharan Africans, W.Eur.: West Eurasians, E.Asi.: Eastern Asians, Aus.: Australasians, Nea.: Neanderthals, Den.: Denisovans.

However, the different approaches to studying reticulate evolution share the same ambition: to represent evolutionary history explicitly, with *phylogenetic networks*. These are simple generalizations of phylogenetic trees, where some nodes—named *reticulations*—are allowed to have multiple direct ancestors [15, 16]. See Fig 1 for two examples of phylogenetic networks, with 3 reticulations each, showing the putative relationships among modern humans and their closest relatives. Networks such as those in Fig 1 are sometimes referred to as “explicit” to distinguish them from other, “data-display”, networks that are not used to represent any particular scenario, but rather to graphically display conflicting phylogenetic signals in the data [15, 16]. (As an example of the latter, see the networks produced by the popular program Neighbor-net [17]). In this paper, we focus on the former type of networks, like those in Fig 1.

Although methods to infer phylogenetic networks are by necessity context-dependent—e.g., gene tree vs. species tree comparisons to study horizontal gene transfers [18], analyses of gene tree frequencies to study inter-specific hybridizations [19, 20], and analyses of SNP allele frequencies to study population admixture [6, 7]—in this paper we examine a component that should be central to all network inference methods: the basic moves that an algorithm should use to explore alternative reticulate scenarios. Given a network such as the one on the left in Fig 1, these moves allow one to generate and evaluate many alternative hypotheses, such as the one on the right in Fig 1. If a network improving the fit with the data is encountered, then the search continues from that network, and is carried on until no more improvements are possible—that is, until a *local optimum* is reached.

These ideas are the natural transposition to phylogenetic networks of what is routinely done for phlogenetic trees, whose reconstruction relies heavily on local search heuristics that explore the space of the possible tree topologies by means of simple rearrangements of their structure. These heuristics have an impressively long tradition (they started appearing in the 1960s [21]) and they have been used in combination with practically all optimization criteria in use, from maximum parsimony and likelihood, to distance-based principles [22, 23]. The best known tree rearrangements are *nearest neighbor interchange* (NNI) and *subtree pruning and regrafting* (SPR). Despite their long history in phylogenetics, the application of topological rearrangement moves within network reconstruction software is very recent (e.g., [24]) and the first mathematically-grounded reflections on how to define these moves to ensure desirable properties are even more recent [25, 26].

In this context, one important difference between trees and networks is that networks can have varying levels of *reticulate complexity*. In the next section, we will see that this term can be formally defined in several equivalent ways—for example, as the number of reticulations in the network. Intuitively, it can be seen as the equivalent of the number of parameters in a statistical model, or as a measure of the explanatory power of the set of networks of that level of complexity: higher complexity—that is, being able to hypothesize more reticulate events—generally allows a better fit with the data [27]. Although alternative measures of network complexity exist (e.g., the *level* of a network, see also the Discussion section), the approach we adopt here is consistent with most approaches to measure model complexity in networks [28, 29]. Interestingly, unless a limit is imposed on reticulate complexity, there exist an infinite number of different networks describing the evolution leading to a given set of sampled taxa, unlike for phylogenetic trees.

Because comparing optimization scores across networks with different complexities may be problematic, in this paper we make a clear distinction between “horizontal” rearrangement moves, which enable the exploration of a “layer” of networks having a fixed reticulate complexity, and “vertical” moves, which allow a change of reticulate complexity, that is, a jump across layers. We will focus on the former, and provide natural definitions of rearrangement moves that generalize the well-known NNI and SPR moves for phylogenetic trees. As we shall show, these moves transform a network of a given reticulate complexity into another network of the same reticulate complexity, and they guarantee that every network of a given complexity is reachable from every other network of the same complexity, within a finite number of moves. Reachability between any two points of a search space is the fundamental property of any rearrangement move that can serve as basis for a search heuristic (see, e.g., the seminal paper on NNI for trees [30]).

**Fig 2:**
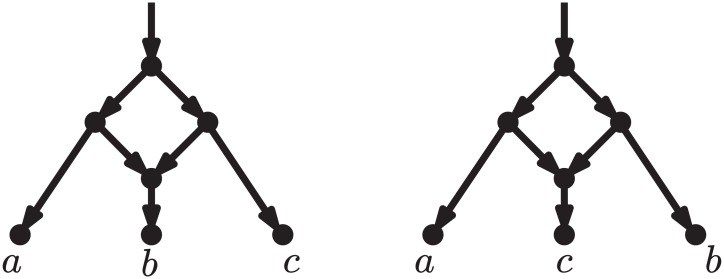
Two networks such that any sequence of rooted LST moves transforming one into the other goes through a tree. LST moves are defined as in Huber et al. [25]. Note that a tree is a less complex model than either of these networks. The rearrangement moves proposed here (rNNI, see below) can transform each of these networks directly into the other (see third line in Fig 8, with *γ* = *c* and *δ* = *b*).

The importance of distinguishing between horizontal and vertical moves lies in the fact that if moves are allowed to change the reticulate complexity of a network, then a sequence of moves transforming one network into the other may have to pass via networks of lower or higher complexity than both *N* and *N*′. This is not optimal: Lower complexity usually implies a lower fit with the data, so if every path from *N* to *N*′ has to pass via networks of lower complexity than both, then even assuming that *N*′ fits the data better than *N*, the search may get stuck before reaching *N*′. (Fig 2, discussed below, shows an example of this for a type of move recently proposed.) Similarly, if every path from *N* to *N*′ contains networks of higher complexity than both, and thus probably of higher fit with the data, it is very hard for a search starting in *N* to ever consider *N*′, as once it moves at a higher complexity, the search will likely stay there. A possible way to deal with these problems is to include in the optimization criterion a regularization term penalizing networks of higher complexity [28, 29].

Two works similar in spirit to the present one have appeared recently [25, 26]. The first of these [25] focuses on level-1 networks (defined in the next section), which are a relatively narrow class of networks, not including, for example, the networks in Fig 1. More importantly, the *local subnetwork transfer* (LST) moves introduced in that paper include both a horizontal and a vertical component, and the results proving the reachability between any two level-1 networks do not distinguish between these components, meaning that any sequence of LST moves transforming a network *N* into another network *N*′ with the same reticulate complexity may have to go through networks of lower (or higher) reticulate complexity. This is precisely what happens for the two networks in Fig 2, where to transform one into the other, the LST moves must pass via a tree. As we explained above, we believe that this is not desirable, because in many realistic scenarios, trees will have a lower fit with the data than either network.

The second paper [26], while it does consider horizontal and vertical moves separately, only focuses on a class of phylogenetic networks that are (*a*) unrooted, and (*b*) such that it is impossible to identify the nodes that represent reticulation events. (We note that *a* does not imply *b*: a definition of unrooted phylogenetic networks in which reticulations are well-determined is recently given by Solís-Lemus and Ané [20].) Rather than representing reticulate evolution explicitly, these networks should be seen as abstract ways to depict evolutionary relationships or, alternatively, as data-display networks. Nevertheless, that paper provides a reachability result between unrooted networks, on which we will rely in some of our proofs.

The rearrangement moves that we define here are named rNNI and rSPR, where the initial “r” denotes the fact that they are defined for rooted, directed phylogenetic networks such as the ones in Fig 1, which are the most intuitive way to represent reticulate evolutionary scenarios explicitly.

The paper is organized as follows: After introducing the necessary mathematical background (in Methods), we give our definition of rNNI moves for networks (generalizing NNI on trees), and prove that any two networks of equal reticulate complexity are mutually reachable by applying rNNI moves (in *rNNI moves on rooted binary networks*). Next, we define rSPR moves for networks (generalizing SPR on trees), and prove that rNNI moves can be seen as “local” rSPR moves (in *rNNI moves as local rSPR moves*). Because of this, reachability trivially extends to rSPR moves. Then, we study properties of the rNNI neighborhood of a network *N*—that is, the set of networks that can be obtained from *N* with just one rNNI move—giving a simple bound on its size (in *Studying the size of the rNNI neighborhood*). Finally, we discuss vertical moves and show that the properties they must have to ensure reachability between any pair of networks (of any complexity) are very minimal (in *Changing the network complexity*). We conclude with a discussion on the relevance of the results obtained to practical search heuristics for phylogenetic network inference (Discussion).

## Methods

In this section we introduce the mathematical preliminaries that are necessary for the rest of the paper.

A graph is *directed* when its edges, called *arcs*, are directed. An arc starting in *u* and ending at *v* is denoted by *uv*; *u* is called the *tail* and *v* the *head* of *uv*. We also call *u* a *parent* of *v*, and *v* a *child* of *u*. The *degree*, *indegree* and *outdegree* of a vertex *v* are the number of arcs incident to *v*, ending at *v* and starting at *v*, respectively (i.e., in a directed graph, the degree is the sum of indegree and outdegree). A directed path from *s* to *t* is called an *s*-*t path*, and is said to be *nonelementary* if it contains at least one vertex other than *s* and *t*. A directed graph is *acyclic* if it contains no directed cycle, that is, no directed path from a vertex to itself. An undirected graph is *connected* if there is a path between every pair of vertices.

Let *X* be a set of taxa. A *rooted (phylogenetic) network* on *X* is a directed acyclic graph with only one indegree-0 vertex, called its *root*, and whose set of outdegree-0 vertices, its *leaves*, is *X*. An *unrooted (phylogenetic) network* on *X* is any connected undirected graph whose set of degree-1 vertices is *X*. Given a rooted phylogenetic network *N*, the *underlying unrooted network* of *N* is the unrooted network obtained from *N* by replacing each arc *uv* in *N* by an undirected edge {*u*, *v*}. A phylogenetic network *N* is a *(phylogenetic) tree* if *N*, or the underlying unrooted network of *N*, does not contain any cycles. A phylogenetic network *N* is *level-1* if its simple cycles, or those of its underlying unrooted network, are pairwise disjoint.

A network is *binary* if any of its vertices has either degree 1 or 3. In the case of binary rooted networks, we also require that the root has outdegree 1. This implies that in a binary rooted network all degree-3 vertices either have indegree 1 and outdegree 2—the *bifurcations*—or indegree 2 and outdegree 1—the *reticulations*. Unless otherwise stated, the rooted networks we consider here do not have parallel arcs, that is, they are not allowed to contain more than one arc of the form *uv*. Note that for a *binary* rooted network on *X* with root *ρ*, its underlying unrooted network is on *X* ∪ {*ρ*}.

**Fig 3:**
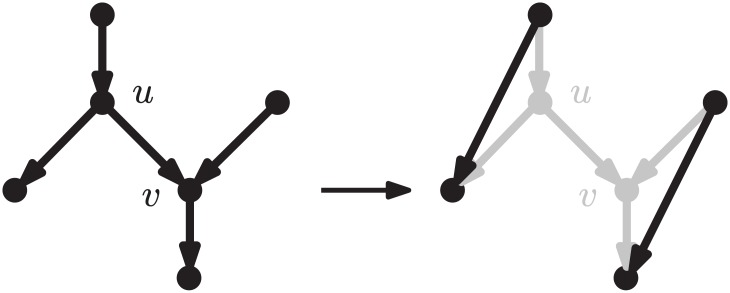
An arc removal.

An *arc removal* in a binary rooted network *N* is the operation of removing from *N* an arc *uv*, where *u* is a bifurcation and *v* is a reticulation, followed by the replacement of *u* with a new arc connecting the parent of *u* with the only remaining child of *u*, and finally by the replacement of *v* with a new arc connecting the only remaining parent of *v* with the child of *v*. See Fig 3 for an illustration of this operation.

We will use repeated arc removals to measure the reticulate complexity of a network (see Proposition 1 below). Note that although this operation can produce directed acyclic graphs with parallel arcs, this is temporary: an additional arc removal applied to one of the two copies of the arc produces a binary rooted network without parallel arcs.

Let *uv* again be an arc connecting a bifurcation *u* to a reticulation *v* in a binary rooted network *N*. Moreover, suppose that *N* contains no nonelementary *u*-*v* path. An *arc flip* consists of replacing such an arc *uv* by the arc *vu*. Note that the conditions imposed above guarantee that an arc flip transforms a binary rooted network into another binary rooted network.

As discussed in the introduction, the rearrangement moves that we define in this paper allow us to explore the space of networks of a fixed reticulate complexity. The following proposition shows that there are several equivalent ways to define the same measure of reticulate complexity.

**Proposition 1.** *Let N*_1_ *and N*_2_ *be two binary rooted networks on X*. *Then the following propositions are equivalent:*

1. *N*_1_ *and N*_2_ *have the same number of reticulations r*.
2. *N*_1_ *and N*_2_ *have the same number of vertices n*.
3. *N*_1_ *and N*_2_ *have the same number of arcs m*.
4. *N*_1_ *and N*_2_ *require the same number of arc removals to be turned into a rooted phylogenetic tree*.

*Proof*. (1 ⇔ 2) In order to prove the equivalence between 1 and 2, we show that for any binary rooted network on *X* the following equation holds:

$$\begin{array}{c} n=2(r+|X|). \end{array}$$

Let *v* be the number of bifurcations and let *I* (resp. *O*) be the sum of indegrees (resp. outdegrees) of the vertices of *N*. We have *I* = *v* + 2*r* + |*X*| and *O* = 1 + 2*v* + *r* (here 1 is for the root). Because *I* = *O*, we have *v* + 2*r* + |*X*| = 1 + 2*v* + *r*, which implies *v* = *r* + |*X*| − 1. Now substitute this in *n* = 1 + *v* + *r* + |*X*| (which expresses the total number of vertices) to obtain the equation above.

(1 ⇔ 3) Note that the number of arcs in a network is equal to the sum of the indegrees (or outdegrees) of its vertices, which we have already derived above. By substituting the expression above for *v* in that for *I* (or *O*), we obtain:

$$\begin{array}{c} m=3r+2|X|-1, \end{array}$$

which shows that two networks on *X* have the same number of reticulations if and only if they have the same number of arcs.

(1 ⇔ 4) We show that any binary rooted network *N* requires exactly *r* arc removals to be turned into a tree. If *N* is not a tree, then it must contain a reticulation *v* such that none of its parents is a reticulation. Then any arc entering *v* can be removed, which reduces the number of reticulations in *N* by one. Thus the number of arc removals that are necessary to turn *N* into a tree is *r*.

Note that in the statistical settings where a network is seen as a probabilistic model, the number of parameters can usually be expressed as a function of the measures above: for example if there is one parameter per arc (usually a branch length) and one parameter per reticulation (e.g., [28]), we have a total of *m* + *r* parameters. In cases such as this one, two networks on the same set of taxa require the same number of parameters if and only if they have the same reticulate complexity.

Our proofs will rely on previous work by Huber and collaborators [26] on NNI moves for unrooted binary networks, defined as follows: Given an unrooted binary network *N* and four distinct vertices (*s*, *u*, *v*, *t*) in *N* such that there exists a path *p* = (*s*, *u*, *v*, *t*) and neither {*s*, *v*} nor {*u*, *t*} are edges of *N*, the NNI move on (*s*, *u*, *v*, *t*) consists in replacing *p* with the path (*s*, *v*, *u*, *t*). In particular we will use the following result.

**Theorem 1.** ([26]). *If N*_1_ *and N*_2_ *are unrooted binary networks on X with the same number of vertices, then there exists a sequence of NNI moves turning N*_1_ *into N*_2_.

## Results

### rNNI moves on rooted binary networks

We say that a rooted phylogenetic network *N* has an arc *on* {*u*, *v*} if it has either the arc *uv* or *vu*.

**Definition 1.** *If a rooted binary phylogenetic network N has four distinct vertices s*, *u*, *v*, *t* *and arcs on* {*s*, *u*}, {*u*, *v*} *and* {*v*, *t*} *but not on* {*u*, *t*} *and* {*s*, *v*}, *then an* rNNI *move consists of replacing the arcs on* {*s*, *u*}, {*u*, *v*} *and* {*v*, *t*} *by arcs on* {*u*, *t*}, {*u*, *v*} *and* {*s*, *v*} *such that:*

1. *the in*- *and outdegrees of s and t are not affected by the move;*
2. *the in*- *and outdegrees of u and v remain at most 2 and*
3. *the obtained network is acyclic*.

*An rNNI move replacing arcs a*_1_, *a*_2_, *a*_3_ *by arcs a*_4_, *a*_5_, *a*_6_ *is denoted by* (*a*_1_, *a*_2_, *a*_3_ → *a*_4_, *a*_5_, *a*_6_). *If Conditions 1 and 2 are satisfied but Condition 3 is not then the move is called a* cycle-creating rNNI move.

Note in particular that an rNNI move may or may not change the orientation of the arc on {*u*, *v*}. Also note that an rNNI move does not change the total degree of any vertex, hence it follows from restrictions 1 and 2 that the network remains binary. Nor does it change the number of vertices of the network, and thus none of the measures of reticulate complexity (see Proposition 1), including the number of reticulations. Moreover, the newly created arcs are necessarily distinct (because all four involved vertices are distinct), and not already present in *N* (because no arcs on {*u*, *t*} and {*s*, *v*} are present in *N*), meaning that an rNNI move cannot produce parallel arcs. Finally we observe that rNNI moves are reversible: if (*a*_1_, *a*_2_, *a*_3_ → *a*_4_, *a*_5_, *a*_6_) is an rNNI move turning *N* into *N*′, then (*a*_4_, *a*_5_, *a*_6_ → *a*_1_, *a*_2_, *a*_3_) is an rNNI move turning *N*′ into *N*.

**Observation 1.** *Applying an rNNI move to a binary rooted phylogenetic network N on X results in another binary rooted phylogenetic network N*′ *on X with the same number of reticulations. Moreover, N can be obtained from N*′ *by an rNNI move*.

**Fig 4:**
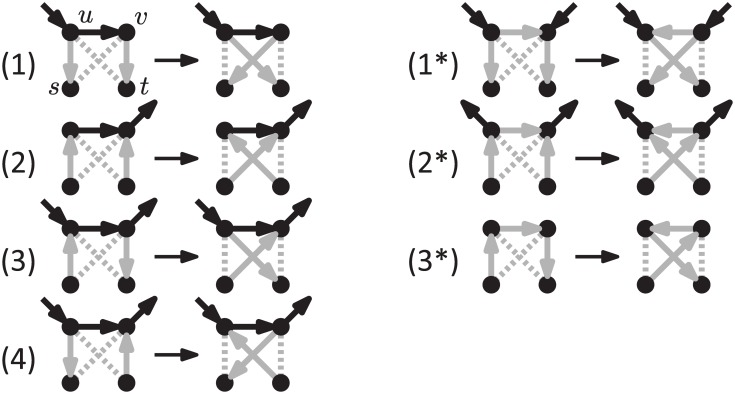
The seven different variants of the rNNI move. Dashed edges indicate that there is no arc between those vertices. Gray arcs are those that change with the move. If vertices have additional incident arcs that are not drawn, then these may be oriented either way. These moves are only valid if the resulting network is acyclic. Note that the difference between (*i*) and (i*) is that (i*) reverses the direction of the *uv* arc.

The rNNI moves can be divided into seven different types, as shown by the following lemma, and illustrated in Fig 4.

**Lemma 1.** *Each rNNI move on rooted binary phylogenetic network N is of one of the following types*.

*(1)* (*us*, *uv*, *vt* → *ut*, *uv*, *vs*) *and there is no s*-*v path in N*;
(1*) (*us*, *uv*, *vt* → *ut*, *vu*, *vs*), *there is no s*-*v path and v is a reticulation in N;*
*(2)* (*su*, *uv*, *tv* → *sv*, *uv*, *tu*) *and there is no u*-*t path in N;*
(2*) (*su*, *uv*, *tv* → *sv*, *vu*, *tu*), *there is no u*-*t path and u is a bifurcation in N;*
*(3)* (*su*, *uv*, *vt* → *sv*, *uv*, *ut*), *u is a reticulation and v a bifurcation in N;*
(3*) (*su*, *uv*, *vt* → *sv*, *vu*, *ut*) *and there is no nonelementary u*-*v path in N;*
*(4)* (*us*, *uv*, *tv* → *vs*, *uv*, *tu*) *and there is no s*-*t path in N*.

*Proof*. An rNNI move assumes the existence of three arcs: one on {*s*, *u*}, one on {*u*, *v*} and one on {*v*, *t*}. We consider the four possible arc orientations for {*s*, *u*} and {*v*, *t*}, while without loss of generality the third arc is fixed as *uv*. For each of these combinations, we consider two possible moves: one leaving the orientation of *uv* unchanged, which gives cases (1)-(4), and one reversing its orientation, which gives cases (1*)-(3*). Note that a (4*) move (*us*, *uv*, *tv* → *vs*, *vu*, *tu*) is not an rNNI, as it would introduce nodes with indegree or outdegree 3. For each of the seven resulting cases, we also provide restrictions that ensure that the conditions given in Definition 1 are satisfied (e.g., *u* has to be a reticulation in (3)). It is tedious but relatively easy to check each of these cases and its associated restrictions.

Note that moves (1*), (2*), (3*) reverse the direction of arc *uv* while moves (1), (2), (3) and (4) do not. Also note that if *N* is a phylogenetic tree, then only the moves of types (1) and (3*) are allowed (as they are the only ones not assuming the presence of a reticulation) and then the rNNI moves defined above coincide with NNI moves on rooted trees.

Recall the definition of arc flips in the *Methods*, as operations that reverse the direction of an arc connecting a bifurcation to a reticulation without introducing cycles. The following three lemmas show some interesting relationships between rooted networks with the same underlying unrooted network, and between rNNI moves on a rooted network and NNI moves on the underlying unrooted network. The proofs of the first two lemmas can be found in the S1 Text.

**Lemma 2.** *Let N be a binary rooted network on X, and let N*′ *be a binary network obtained by applying an arc flip to N. Then, unless N and N*′ *are the same network (that is, they are isomorphic), N can be turned into N*′ *in exactly two rNNI moves*.

**Lemma 3.** *Let N be a binary rooted phylogenetic network and let N_u_* *be its underlying unrooted network. If an unrooted network* $N_{u}^{\prime }$ *can be obtained by applying a single NNI move to N_u_*, *then there exists a sequence of rNNI moves turning N into a network N*′ *that has* $N_{u}^{\prime }$ *as its underlying unrooted network*.

**Lemma 4.** *If N and N*′ *are binary rooted level-1 phylogenetic networks on X with the same underlying unrooted network, then there exists a sequence of arc flips turning N into N*′.

*Proof*. The gist of the proof is the following: because *N* and *N*′ have the same underlying unrooted network, then their cycles only differ by paths that are oriented in opposite directions in each of the networks. A flipping applied to each of the arcs in these paths transforms one of the networks into the other one. However, to be valid, these arc flips must be performed in a specific order, as we now describe.

For any node *u* in *N*, let *d*_*N*_(*u*) be the length of a longest path from the root of *N* to *u*. We say that an arc *xy* of *N* is *incorrectly oriented* if *N*′ has the arc *yx* and *correctly oriented* if *N*′ has the arc *xy*. If *N* ≠ *N*′ then *N* has at least one incorrectly oriented arc. Let *uv* be an incorrectly oriented arc of *N* such that there is no *u*′*v*′ that is incorrectly oriented and *d*_*N*_(*u*′) > *d*_*N*_(*u*).

First, *v* is a reticulation in *N* since if it were a bifurcation, for our choice of *u*, *v* would have two correctly-oriented outgoing arcs in *N* and hence outdegree 3 in *N*′, and if *v* were a leaf, then *N*′ would not be on the same set of taxa *X* as *N*. Second, *u* is a bifurcation in *N* since if it were a reticulation *N* would not be level-1, while if it were the root of *N*, then *N*′ would have *u* among its leaves, implying that *N* and *N*′ would not be on the same set *X*.

Now, we want to prove that there is no nonelementary *u*-*v* path in *N*. Suppose that there exists one. Then this path has at least one incorrectly oriented arc *u*′*v*′, otherwise *N*′ would contain a cycle. Then, because of our choice of *u*, we know that *u*′ = *u*. Now, notice that *v*′ cannot be a reticulation, since otherwise *N* would not be level-1. Again, for our choice of *u*, both outgoing arcs of *v*′ in *N* are correctly oriented. Then *uv*′ cannot be incorrectly oriented unless *v*′ has outdegree 3 in *N*′, which is impossible.

Hence, there is no nonelementary *u*-*v* path in *N*. Therefore, we can perform an arc flip on *uv* and reduce the number of incorrectly-oriented arcs by one. We repeat this until there are no incorrectly-oriented arcs left.

As we now show, the three lemmas above allow us to prove the restriction of our main result on rNNI moves to level-1 networks. Note that in the theorem below we do not require intermediate networks to be level-1.

**Theorem 2.** *If N and N*′ *are binary rooted level-1 phylogenetic networks on X with the same number of reticulations, then there exists a sequence of rNNI moves turning N into N*′.

*Proof*. By Theorem 1, there exists a sequence of unrooted NNI moves that turns the underlying unrooted network of *N* into the underlying unrooted network of *N*′ (by Proposition 1 these unrooted networks have the same number of vertices). Hence, by Lemma 3, there exists a sequence of rNNI moves that turns *N* into a network *N*′′ (on *X*) that has the same underlying unrooted network as *N*′. By Lemma 4, there exists a sequence of arc flips turning *N*′′ into *N*′, which by Lemma 2 can be reproduced by a sequence of rNNI moves. Together, this gives a sequence of rNNI moves turning *N* into *N*′.

**Fig 5:**
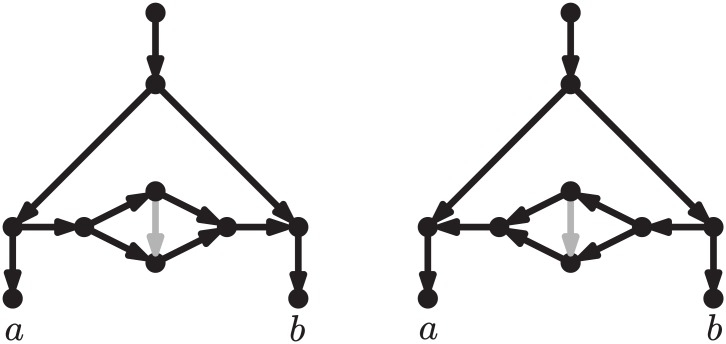
Lemma 4 does not hold for general networks. Two rooted networks with the same underlying unrooted network that are not reachable from one another by performing a sequence of arc flips. The only arc flips that can be applied are to the gray arcs, as the reversal of any other arc produces a network that either is nonbinary or contains a cycle.

Interestingly, Lemma 4 does not hold for networks that are not level-1, as shown in Fig 5. This means that in order to generalize Theorem 2, we need to adopt a more complex approach. These observations prompt the next definition.

**Definition 2.** *A binary rooted phylogenetic network N on X is called* flip-friendly *if it can be transformed into any other binary rooted network N*′ *on X with the same underlying unrooted network as N by repeatedly applying arc flips*.

Note that if *N* is flip-friendly, then every network with the same underlying unrooted network as *N* is also flip-friendly. Although not every binary rooted network is flip-friendly (e.g., Fig 5), the following lemma—whose proof can be found in the S1 Text—shows that there are flip-friendly networks at every level of reticulate complexity.

**Lemma 5.** *For any nonempty X and r ≥ 1, there exists at least one flip-friendly binary rooted network on X with r reticulations*.

This result allows us to prove that any two binary rooted networks of equal reticulate complexities are reachable from one another via rNNI moves, by using a slightly different approach than that employed to prove Theorem 2.

**Theorem 3.** *Let N*_1_ *and N*_2_ *be two binary rooted phylogenetic networks on X with the same number of reticulations r. Then there exists a sequence of rNNI moves turning N*_1_ *into N*_2_.

*Proof*. Let *N*_*F*_ be a flip-friendly binary rooted network on *X* with *r* reticulations, which exists by Lemma 5, and let $N_{F}^{u}$ be its underlying unrooted network. Also let $N_{1}^{u}$ be the underlying unrooted network of *N*_1_. By Theorem 1, there exists a sequence of NNI moves transforming $N_{1}^{u}$ to $N_{F}^{u}$, and thus by Lemma 3, there is a sequence *S*_1_ of rNNI moves transforming *N*_1_ into a network $N_{1}^{\prime }$ such that $N_{1}^{\prime }$ has $N_{F}^{u}$ as underlying unrooted network. By the same argument, there is a sequence *S*_2_ of rNNI moves transforming *N*_2_ into a network $N_{2}^{\prime }$ also having $N_{F}^{u}$ as underlying unrooted network. Because *N*_*F*_ is flip-friendly and $N_{1}^{\prime }$ and $N_{2}^{\prime }$ are binary rooted networks on *X* with the same underlying unrooted network as *N*_*F*_, *N*_*F*_ can be turned into $N_{1}^{\prime }$ and $N_{2}^{\prime }$ by only using arc flips. But then, as arc flips are reversible, $N_{1}^{\prime }$ can be turned into $N_{2}^{\prime }$ by a sequence of arc flips, which by Lemma 2 corresponds to a sequence *S*_*flip*_ of rNNI moves. Then one can obtain *N*_2_ from *N*_1_ by first applying *S*_1_ to obtain $N_{1}^{\prime }$, then applying *S*_*flip*_ to obtain $N_{2}^{\prime }$, and finally applying *S*_2_ in reverse order to obtain *N*_2_.

An interesting consequence of Theorem 3 is that our definition of rNNI moves induces natural metrics over the spaces of the rooted binary networks of fixed reticulate complexity: if we let *N*_1_ and *N*_2_ be two binary rooted phylogenetic networks on *X* with the same number of reticulations, their *rNNI distance* can be defined as the minimum number of rNNI moves required to transform *N*_1_ into *N*_2_ (or vice versa, because of the reversibility of the moves). It is easy to see that this definition satisfies the conditions for a metric.

### rNNI moves as local rSPR moves

In this section, we will give a natural definition of SPR moves on binary rooted networks (rSPR), and show that the rNNI moves defined above have a very simple interpretation as rSPR moves that regraft an arc “locally”.

**Definition 3.** *Let xz*, *zy* *and* *x*′*y*′ *be three arcs in a rooted binary phylogenetic network N*, *such that x*′ ≠ *z* ≠ *y*′ *and none of x*′*z*, *zy*′ *and* *xy is a arc of N*. *Then an* rSPR *move consists of replacing the arcs xz*, *zy* *and x*′*y*′ *with x*′*z*, *zy*′ *and* *xy*, *under the condition that the resulting network is acyclic. Such a move is denoted by* [*xz*, *zy*, *x*′*y*′ → *x*′*z*, *zy*′, *xy*]. *Arcs xz* *and* *zy* *are called the* donor *arcs and x*′*y*′ *is the* recipient *arc*.

**Fig 6:**
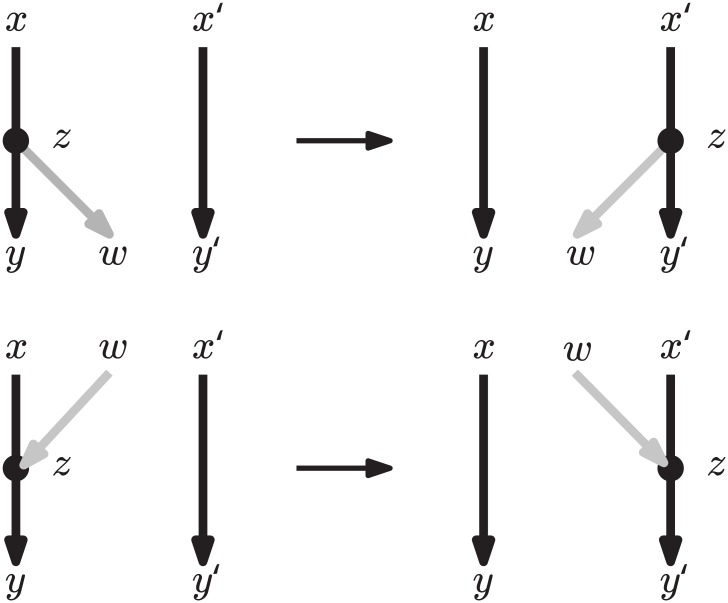
Illustration of rSPR moves. The donor arcs (*xz* and *zy*) and the recipient arc (*x*′*y*′) are in black, while the arc whose head or tail is moved is drawn in grey.

Note that vertex *z* is either the tail of an arc *zw* or the head of an arc *wz*. Informally, an rSPR move can be described as moving (or “regrafting”) the tail or the head of this arc from the donor “arc” *xy*, to the recipient arc *x*′*y*′ (see Fig 6). We call the former type of rSPR move *tail-moving*, and the latter *head-moving*. As stated, such moves are only allowed if they do not create cycles in the network. Note that when applied to a phylogenetic tree, the rSPR moves can only be tail-moving; they then coincide with the rooted SPR operations commonly defined on rooted trees [31, 32]. The name rSPR is meant to stand for *rooted subnetwork pruning and regrafting*, where the subnetwork being affected can be identified as the one consisting of all descendants of the pruned arc in a tail-moving rSPR, or of all of its ancestors in a head-moving rSPR move.

The definition above implies that the newly created arcs {*x*′*z*, *zy*′, *xy*} are necessarily distinct and not already present in *N*, meaning that an rSPR move cannot create parallel arcs. Moreover, an rSPR move does not change the number of vertices of the network (and thus none of the measures of reticulate complexity in Proposition 1), nor the indegree or outdegree of any vertex. Thus an rSPR move always turns a binary rooted network into another binary rooted network, and, like rNNI moves, it is easy to see that rSPR moves are reversible.

**Observation 2.** *Applying an rSPR move to a binary rooted phylogenetic network N on X results in another binary rooted phylogenetic network N*′ *on X with the same number of reticulations. Moreover, N can be obtained from N*′ *by an rSPR move*.

We now provide the conditions that determine whether a candidate rSPR move creates a cycle in a network.

**Lemma 6.** *Similarily to Definition 3, let xz*, *zy* *and x*′*y*′ *be three arcs in a rooted binary phylogenetic network N*, *such that x*′ ≠ *z* ≠ *y*′ *and none of x*′*z*, *zy*′ *and xy is an arc of N*. *Furthermore, let w be the vertex adjacent to z in N that is neither x nor y, and let N*′ *be the directed graph obtained from N by replacing the arcs in* {*xz*, *zy*, *x*′*y*′} *with those in* {*x*′*z*, *zy*′, *xy*} *(see* Fig 6 *for an illustration)*.

1. *If the move is tail-moving (i.e. zw is an arc of N), N*′ *is acyclic if and only if there is no w-x*′ *path in N*.
2. *If the move is head-moving (i.e. wz is an arc of N), N*′ *is acyclic if and only if there is no y*′ *-w path in N*.

*Proof*. The *only if* parts are trivial, as it is easy to check that the indicated paths imply a cycle in *N*′. As for the *if* part, the fact that *N* is acyclic implies that there cannot be any cycles in *N*′ not containing *zw* (in the tail-moving case) or *wz* (in the head-moving case). But then the existence of a cycle in *N*′ would imply the existence of a *w*-*x*′ path, or of a *y*′-*w* path, respectively, in *N*′, and therefore in *N*.

NNI moves for phylogenetic trees are often viewed as SPR moves that regraft a subtree onto an edge that is incident to the edge from which the subtree was initially pruned [33]. This observation prompts the following definition.

**Definition 4.** *An* rSPR_1_ *move is an rSPR move where the recipient arc is incident with one of the donor arcs*.

**Fig 7:**
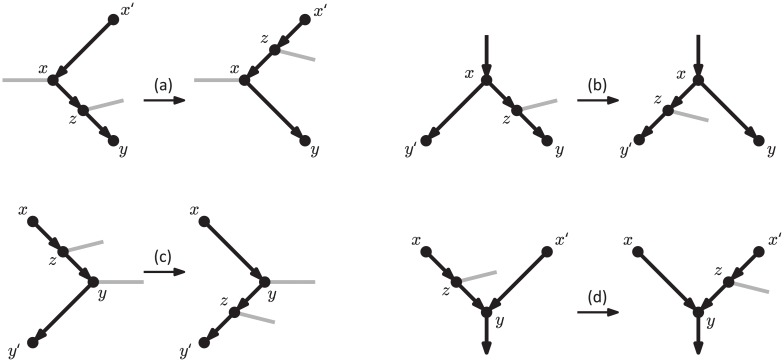
Illustration of rSPR_1_ moves. Vertex *z* and its incident arc can only be regrafted onto: (a) an arc entering *x*, (b) an arc exiting *x*, (c) an arc exiting *y*, (d) an arc entering *y*. Grey arcs are the ones whose direction is undetermined.

Note that because of the requirement in Definition 3 that the recipient arc *x*′*y*′ cannot be incident to *z*, an rSPR_1_ move can only regraft vertex *z* and its incident arc to one of four possible recipient arcs (see Fig 7).

We can now state the main result of this section. Its relatively tedious proof—which can be found in the S1 Text—consists of showing that each of the four types of rSPR_1_ moves in Fig 7 is in fact an rNNI move, and, conversely, each of the seven rNNI types in Lemma 1 can be reproduced with a single rSPR_1_ move.

**Theorem 4.** *Let N and N*′ *be binary rooted networks. Then, N can be turned into N*′ *with one rNNI move if and only if N can be turned into N*′ *with one rSPR*_1_ *move*.

Theorem 4 implies that every rNNI move is also an rSPR move, and every sequence of rNNI moves (e.g., that in Theorem 3) is also a sequence of rSPR moves. If we define the *rSPR distance* of two networks as the minimum number of rSPR moves to transform one network into the other, we then have the following result.

**Corollary 1.** *Let N*_1_ *and N*_2_ *be two binary rooted networks on X with the same number of reticulations. Then there exists a sequence of rSPR moves turning N*_1_ *into N*_2_. *Moreover, the rSPR distance between N*_1_ *and N*_2_ *is at most equal to their rNNI distance*.

### Studying the size of the rNNI neighborhood

The two previous subsections provide two alternative definitions for rNNI moves. One important aspect that they have in common is that one arc in the starting network *N* is “central” in both definitions: *uv* in the original definition (Def. 1) and the donor arc incident to the recipient arc in the definition of rSPR_1_ moves (e.g., arc *xz* in Fig 7(a); Def. 4). We say that the rNNI move is *around* this arc.

We can list the different networks that can be reached from a network *N* with one rNNI move—that is, the *rNNI neighborhood* of *N*—by considering one internal arc of *N* at a time, and then by enumerating the networks *N*′ that can be obtained with one rNNI move around that arc. This is the approach we take to prove the following bound on the size of the rNNI neighborhood.

**Proposition 2.** *Let N be a binary rooted network. Within N, let e_BB_ denote the number of arcs from a bifurcation to a bifurcation, e_BR_ the number of arcs from a bifurcation to a reticulation, e_RB_ the number of arcs from a reticulation to a bifurcation, and e_RR_ the number of arcs from a reticulation to a reticulation. Then, the number of different binary rooted networks that can be obtained from N by one rNNI move is at most* 2(*e_BB_* + *e_RR_*) + 3*e_BR_* + 4*e_RB_*.

**Fig 8:**
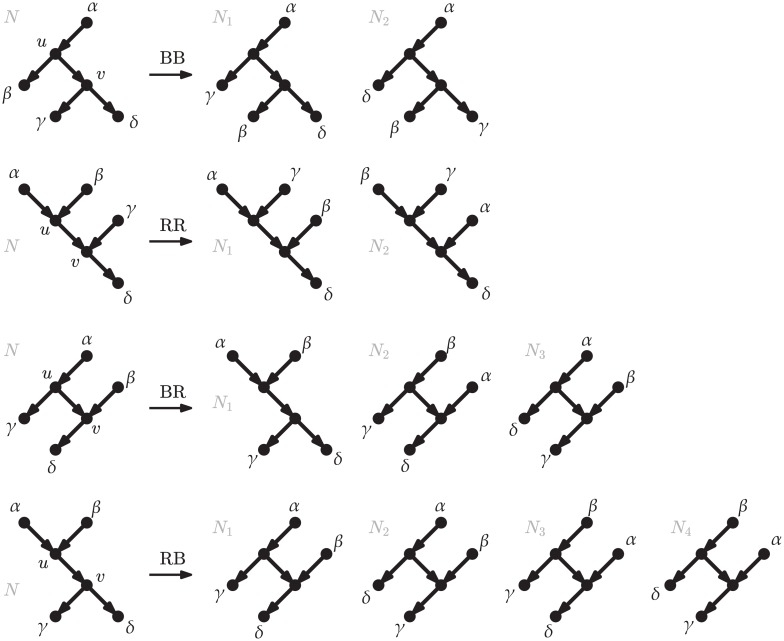
rNNI moves around the different types of internal arcs. For each type (BB, RR, BR, RB), we list the networks that can be obtained by performing an rNNI around that an arc of that type. The four types of arc are named on the basis of *u* and *v* being a bifurcation (B) or a reticulation (R). If some of the vertices in the drawing are not distinct (e.g., if *α* = *γ* in a move of type RR), or if a *γ*-*β* path exists in a move of type BR, then some of the moves above may not be applicable. See the proof of Proposition 2 (in the S1 Text) for details.

Although we refer the reader to the S1 Text for a detailed proof of Proposition 2, we give a brief outline here: because every rNNI move applied to *N* must be around some arc *uv* in *N*, where each of *u* and *v* can either be a bifurcation or a reticulation, rNNI moves can be divided into four cases: those around an arc from a bifurcation to a bifurcation (case BB), from a reticulation to a reticulation (case RR), and those around and arc connecting a bifurcation and a reticulation (in any order, cases BR and RB). By considering these four cases, it is easy to see that for cases BB and RR at most 2 other network topologies can be obtained from *N*, while for cases BR and RB at most 3 and 4 networks can be obtained, respectively, which gives the claimed bound. See Fig 8 for an illustration of these four cases.

**Fig 9:**
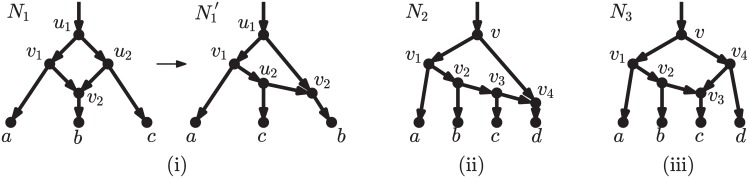
Reasons why the bound of Prop. 2 is not tight. (i) rNNI moves around different arcs may give the same network: the rNNI type-(1) move (*u*_1_*u*_2_, *u*_1_*v*_1_, *v*_1_*v*_2_ → *u*_1_*v*_2_, *u*_1_*v*_1_, *v*_1_*u*_2_) and the rNNI type-(2) move (*u*_1_*u*_2_, *u*_2_*v*_2_, *v*_1_*v*_2_ → *u*_1_*v*_2_, *u*_2_*v*_2_, *v*_1_*u*_2_) on *N*_1_ both give the network $N_{1}^{\prime }$. (ii) Some of the moves drawn in Fig 8 are not viable rNNI moves: no move of type BR around arc *vv*_4_ of *N*_2_ is allowed, because of the presence of a *v*_1_-*v*_3_ path, which would cause the resulting network to contain a cycle. (iii) The bound of Prop. 2 is tight for some networks: the size of the rNNI neighborhood of network *N*_3_, equal to 12, coincides with the bound.

When *N* is a tree, only case BB is applicable and the bound above gives twice the number of internal arcs, coinciding with the classic result on the size of the NNI neighborhood for phylogenetic trees. We note that rNNI moves around different arcs may result in the same network (see, e.g., Fig 9(i)), which means that if we consider one arc at a time and enumerate all networks that can be obtained with one rNNI move around that arc, we may end up listing the same network twice. An extreme case of this situation is given in the S1 Text, where we show a family of networks whose neighborhood has logarithmic size in the number of arcs, whereas the upper bound given above is linear in the number of arcs.

It may be difficult to derive the exact size of the rNNI neighborhood, unless important limitations on the structure of the network are imposed. For example, in the context of unrooted networks, Huber and colleagues derived an exact formula for the size of the NNI neighborhood (see Theorem 3 of [25]), but only for the restricted subclass of unrooted level-1 networks: distinguishing distinct cases for cycles of length 3 or 4 allows them to deal with cases such as the one of Fig 9(i). However, on rooted networks, acyclicity constraints also need to be taken into account, and do not permit a simple formula even for level-1 networks, like *N*_2_ in Fig 9(ii). Note that the upper bound of Proposition 2 is tight in some cases, e.g. for *N*_3_ of Fig 9(iii), as detailed in S1 Text. Interestingly, *N*_2_ and *N*_3_ in Fig 9 have the same underlying unrooted network, and the same values for *e*_*BB*_, *e*_*BR*_, *e*_*RB*_ and *e*_*RR*_, yet different sizes of rNNI neighborhoods, showing that any exact formula for the size of the rNNI neighborhood must depend on parameters of the network other than those used here or by Huber et al. [25].

### Changing the network complexity

Both topological rearrangements defined above only permit the exploration of the set of networks of a fixed reticulate complexity, where the complexity of a phylogenetic network can be defined in any of the ways specified by Proposition 1. In some applications this may be sufficient, for example to tackle optimization problems where network reconstruction is constrained to networks with a prespecified number of reticulations. In many cases, however, one would like to be able to move across spaces of networks of different complexities, so that *any* two rooted binary networks are reachable from one another.

In order to do this, consider a pair of rearrangement moves $C^{+}$, $C^{-}$ with the following properties: $C^{+}$ can transform any rooted binary network *N* on *X* into a number of rooted binary networks on *X* with a level of complexity immediately higher than *N* (i.e., the transformed network has one more reticulation and two more vertices). Conversely, $C^{-}$ maps any rooted binary network *N* that is not a phylogenetic tree to some rooted binary networks on the same set of taxa, and with a level of complexity immediately lower than *N*. We call such a pair of rearrangement moves a *complexity-changing rearrangement pair*. It is easy to see that a number of natural rearrangement pairs can be defined. For example, $C^{-}$ and $C^{+}$ can be defined as arc removals (see the Methods) and arc insertions, respectively, or as rooted versions of the Δ^−^ and Δ^+^ moves by Huber et al. [26]. Precise definitions will be given below. The following proposition is a direct consequence of Theorem 3.

**Proposition 3.** *Let* $C^{+}$ *and* $C^{-}$ *be a complexity-changing rearrangement pair. If N*_1_ *and N*_2_ *are rooted binary networks on X, then there exist: (1) a sequence of rNNI and* $C^{+}$ *moves connecting N*_1_ *and* *N*_2_ *and (2) a sequence of rNNI and* $C^{-}$ *moves connecting N*_1_ *and N*_2_.

*Proof*. Without loss of generality, let *N*_1_ have fewer reticulations than *N*_2_ (or the same number). One can transform *N*_1_ into *N*_2_ by applying $C^{+}$ moves until obtaining a network with the same number of reticulations as *N*_2_, which then, thanks to Theorem 3, can be turned into *N*_2_ using only rNNI moves. In the same way, *N*_2_ can be transformed into *N*_1_ by using $C^{-}$ moves until obtaining a network with the same complexity as *N*_1_, followed by rNNI moves.

Note that the proposition above makes very few assumptions on the chosen complexity-changing rearrangement pair. Namely, it holds even if $C^{+}$ and $C^{-}$ are not the reverse of each other, which could happen for example if we define $C^{+}$ so that it maps every network with *r* reticulations to the same single network with *r* + 1 reticulations. However these kinds of moves are unlikely to have any relevance in practice.

As an example of a realistic complexity-changing rearrangement pair, consider defining $C^{-}$ as the arc removals that do not create parallel arcs, and $C^{+}$ as their reverse operation. We refer to such $C^{+}$ moves as *arc insertions*. They simply consist of choosing two distinct arcs *a*, *a*′ in the network—with *a*′ not ancestral to *a*—followed by creating two new vertices *u*, *v* that subdivide *a* and *a*′, respectively, and finally by adding a new arc *uv*. A variation of arc insertion was first proposed by Jin et al. [34], where further constraints are imposed on the arcs *a*, *a*′ that can be connected.

**Proposition 4.** *Let N*^−^ *and N*^+^ *be binary rooted networks. N*^+^ *can be obtained by performing an arc insertion on N*^−^ *if and only if N*^−^ *can be obtained by performing an arc removal on N*^+^.

*Proof*. If *N*^+^ is obtained from *N*^−^ by inserting arc *uv*, then clearly *u* is a bifurcation and *v* is a reticulation. We can then apply an arc removal to *uv* to obtain *N*^−^ from *N*^+^. If *N*^−^ is obtained from *N*^+^ by removing arc *uv*, then let *a* and *a*′ be the arcs in *N*^−^ that replace *u* and *v*, respectively. Clearly, *a*′ cannot be ancestral to *a*, as otherwise there would be a *v*-*u* path in *N*^+^, which would make it contain a cycle. Applying an arc insertion between *a* and *a*′ in *N*^−^, which amounts to re-inserting *uv* in *N*^−^, results in *N*^+^.

Another example of complexity-changing rearrangement pair can be given by adapting to the rooted case the Δ^+^ and Δ^−^ moves defined by Huber et al. [26] for unrooted networks: we simply define a rooted Δ^+^ move as an arc insertion (as defined above) between two arcs *a*, *a*′ that are incident. Its reverse, the rooted Δ^−^ move, is any arc removal that is applied to an arc whose endpoints are separated by two incident arcs. In the same way as rNNI moves can be seen as “local” rSPR moves, the Δ^+^ and Δ^−^ moves are “local” arc insertions and arc removals.

## Discussion

In this paper, we have generalized to rooted phylogenetic networks the best-known tree rearrangement moves: *nearest neighbor interchange* (NNI) and *subtree pruning and regrafting* (SPR). The new moves, which we call rNNI and rSPR, transform a network of a given reticulate complexity into another network of the same complexity, and they guarantee that every network of a given complexity is reachable from every other network of the same complexity, within a finite number of moves (Theorem 3).

Here, reticulate complexity is measured in terms of number of reticulations, or, equivalently, number of vertices in the network. This measure of complexity is the one that most closely models the “explanatory power” of a network, as it is often directly related to the number of free parameters in a network model (e.g., branch lengths and inheritance proportions at each reticulation [28]). We note that another measure of complexity is often adopted in the computational phylogenetics literature: the *level* of the network [35, 36]. This measure, however, essentially has a motivation in terms of computational complexity, rather than in terms of ability to fit the data. It is related to the algorithmic efficiency of solving some fundamental problems on the network—most notably, for the purposes of this paper, that of evaluating a network under a number of optimization criteria (e.g. [34, 37]).

Another choice we have made in this paper is to not allow multi-edges, or parallel arcs, in the networks we consider, on the assumption that they are difficult to reconstruct from real data. In a number of applications, this may not be true [38, 39]. The rearrangement moves that we define here are easy to adapt so that they can deal with networks containing parallel arcs: for example, in the definition of rSPR moves, it suffices to remove the condition that “none of *x*′*z*, *zy*′ and *xy* is an arc of *N*” (which immediately also determines a definition of rNNI as rSPR_1_).

In addition to the “horizontal” moves above, which enable full exploration of a layer of networks of fixed complexity, this paper also provides the basic ideas on how to switch across spaces of networks of different complexities, via “complexity-changing” or “vertical” moves. Although very little assumptions on vertical moves are needed if we only wish to ensure reachability of any network from any other network (Proposition 3), it is likely that in practice the choice of adequate vertical moves will be important.

In practical search heuristics, it seems reasonable to only increase the complexity of a candidate network (via a vertical move) once a layer of networks of equal complexity has been sufficiently explored via horizontal moves. If we follow this guideline, then the obvious way to proceed would be to first look for the best tree with respect to the data and the chosen optimization criterion, then the best network with one reticulation, then the best network with two reticulations, and so on. This is indeed a common approach in practice [19, 29, 34, 40], and produces a list of networks of increasing complexity and fit to the data. In order to choose between these networks, techniques for model selection are often advocated: for example the Akaike (AIC) or the Bayesian information criterion (BIC) [24, 28, 29], or nonparametric techniques such as cross-validation [24]. The fact that our horizontal and complexity-increasing moves $C^{+}$ are enough to go from any starting tree to any network (Proposition 3) provides a theoretical basis for this approach: no network inferred early on in the list precludes the inference of another network at a later stage.

Another aspect that deserves to be discussed is the locality of the proposed rearrangement moves. Clearly, rNNI and rSPR moves provide different degrees of locality for horizontal moves, and Δ^+^/Δ^−^ moves and arc insertions/removals do the same for vertical moves. Recall that the neighborhood of a network *N* with respect to a rearrangement move is the set of networks that can be reached with one move from *N*. Choosing moves that are less local implies increasing the sizes of the neighborhoods, which means fewer local optima, thus potentially more accurate reconstructions. On the other hand, local moves are often considered to lead to fewer computations, as fewer neighbors need to be considered at each iteration of the search heuristic; this is counterbalanced by the fact that, typically, more iterations are needed to find an optimum (see Corollary 1). In practice adapting the degree of locality is a question of craftsmanship, and the best practices may be context-dependent. For example, the locality of the moves can change during the search, typically increasing with later iterations. Interestingly, the fact that rNNI moves can be seen as rSPR_1_ moves immediately suggests that several intermediate degrees of locality can be achieved by defining rSPR_*k*_ moves allowing a maximum distance *k* between the recipient and the donor arcs in an rSPR. Similarly, vertical moves that are intermediate between Δ^+^/Δ^−^ moves and arc insertions/removals can be defined by bounding the distance between the endponts of the arc being inserted/removed.

Our work provides a theoretical basis to analyse the search strategy implemented in the most popular program for network reconstruction, PhyloNet [41]. In the implementation described by Yu et al. [24], the search proceeds by randomly generating networks produced by horizontal or vertical moves that are at the non-local end of the spectrum of the moves described here (they are essentially equivalent to rSPR moves and arc insertions/removals, although parallel arcs seem to be allowed there). Note that horizontal and vertical moves can occur during the search in any order. Our results imply that PhyloNet is able to reach any binary rooted network from any other binary rooted network—unsurprisingly, given the large size of the neighborhoods considered in its search. Proposition 3 shows that in fact reachability can be assured even under much more local moves. Future work on practical heuristics for network reconstruction will be likely inspired by common practices for tree reconstruction implemented by popular software such as PhyML [42] and RAxML [43]. In particular, it should be possible to speed up the evaluation (i.e. the calculation of the optimization score) of the networks in the neighborhood of a network that has already been evaluated, by identifying the parts of the computation that do not need to be repeated.

Another direction for future research is to constrain horizontal rearrangement moves so as to preserve not only reticulate complexity, but also the level of the network. Given that the level is often related to the computational complexity of computing the optimization score of a network (e.g., for parsimony [37], and for likelihood [34]), it would be useful to keep the level bounded during the local search. An interesting open question is to determine the maximum level reached by intermediate networks when transforming a level-*k* network into another level-*k* network with the same number of reticulations via rNNI moves or rSPR moves. An advantage of rSPR moves is that, given the larger sizes of their neighborhoods, they may be able to to avoid high-level intermediate networks.

Finally, in this paper we have not tackled natural questions related to the metrics induced by the moves defined here—such as the maximum distance between networks—which for trees have been studied in depth [30, 44–46]. From an algorithmic standpoint, we remark that because our rNNI and rSPR distances reduce to well-known distances on phylogenetic trees, all the known hardness results on computing such distances on trees extend to our distances [32, 47].

## Supporting information

S1 Text Supporting information: Proofs omitted from the main text. This document provides the proofs of Lemmas 2, 3, 5, Theorem 4, and Proposition 2. It ends with a few remarks on the size of rNNI neighborhoods.
(PDF)
