# Supplementary material for: Rearrangement moves on rooted phylogenetic networks
Source: PLoS Comput Biol. 2017 Aug 1;13(8):e1005611. doi: 10.1371/journal.pcbi.1005611 (PMC5557604; doi:10.1371/journal.pcbi.1005611)
Supplement: S1 Text — This document provides the proofs of Lemmas 2, 3, 5, Theorem 4, and Proposition 2. It ends with a few remarks on the size of rNNI neighborhoods. (PDF) [file pcbi.1005611.s001.pdf]

Supporting Text S1: proofs omitted from the main text

Here, we provide full proofs for Lemmas 2, 3, 5, Theorem 4, and Proposition 2. We conclude with a few remarks on the size of rNNI neighborhoods.

Lemma 2. *Let N be a binary rooted network on X, and let N' be obtained by applying an arc flip to N. Then, unless N and N' are the same network (that is, they are isomorphic), N can be turned into N' in exactly two rNNI moves.*

Proof. Let uv be the arc being flipped in N. First suppose that the parent s of u and the parent $t \neq u$ of v are distinct vertices. Then we apply a type-(2) rNNI move $(su, uv, tv \to sv, uv, tu)$. This is allowed because if there were a u-t path, there would be a nonelementary u-v path in N, which is not the case by the assumption that arc uv can be flipped. Now we can apply a type-(2^*) move $(sv, uv, tu \to su, vu, tv)$, because no u-s path can exist in N. The net effect of these two moves is that arc uv is reversed to vu, see Figure S1.

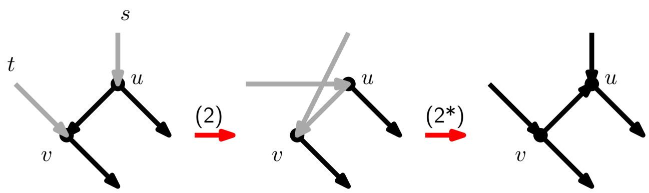

Fig S1. Reversing an arc uv when u and v have different parents.

Now suppose that u and v have a common parent p but the child $\hat{s} \neq v$ of u and the child $\hat{t}$ of v are distinct vertices. Then we apply a type-(1) rNNI move $(u\hat{s}, uv, v\hat{t} \to u\hat{t}, uv, v\hat{s})$. This is allowed because if there were an $\hat{s}v$ path in N, this path would need to pass through p, and hence imply the existence of a directed cycle in N. Now we can apply a type-(1^*) move $(u\hat{t}, uv, v\hat{s} \to u\hat{s}, vu, v\hat{s})$, because no $\hat{t}$-v path can exist in N. The net effect of these two moves is that arc uv is reversed to vu, see Figure S2.

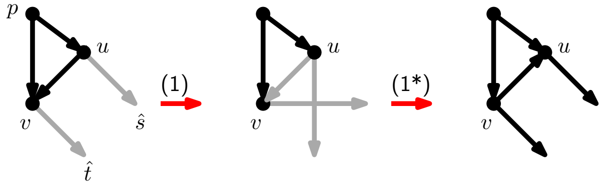

Fig S2. Reversing an arc uv when u and v have a common parent but different children.

If we are in neither of the previous cases, u and v have a common parent p and a common child c. But then it is easy to see that that N and N' are isomorphic (just map u to v and viceversa), meaning that no rNNI move is needed to turn N into N'. □

Lemma 3. *Let N be a binary rooted phylogenetic network and let N_u be its underlying unrooted network. If an unrooted network N'_u can be obtained by applying a single NNI move to N_u, then there exists a sequence of rNNI moves turning N into a network that has N'_u as its underlying unrooted network.*

Proof. There are four ways in which the edges affected by the NNI move can be oriented in N, see the four networks to the left in Fig. 4 in the main text. In each case, there is at

least one move that satisfies conditions 1 and 2 of Def. 1 (the degree conditions). Hence, there exists a (possibly cycle-creating) rNNI move turning N into N' such that N' has N'_u as its underlying unrooted network. However, N' may contain a directed cycle. Note that a move of type (3) cannot create a directed cycle. Moreover, if the move is of type (1^*) or (2^*), then it can be replaced by a move of type (1) or (2) without changing the underlying unrooted network. Hence, we only need to consider moves of types (1),(2),(3^*) and (4). These moves can create a directed cycle in the following cases:

(1) $(us, uv, vt \to ut, uv, vs)$ and there is an s-v path in N;

(2) $(su, uv, tv \to sv, uv, tu)$ and there is a u-t path in N;

(3^*) $(su, uv, vt \to sv, vu, ut)$ and there is a nonelementary u-v path in N;

(4) $(us, uv, tv \to vs, uv, tu)$ and there is a s-t path in N.

For each of these cases, we show that an acyclic network N'' with the same underlying unrooted network as N' can be obtained from N by applying a sequence of rNNI moves.

Case (1). $(us, uv, vt \to ut, uv, vs)$ and there is a s-v path in N, see Figure S3.

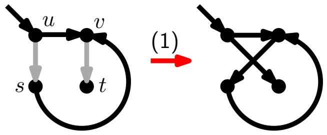

Fig S3. A cycle-creating rNNI move of type (1).

The s-v path must contain at least one internal vertex since N does not contain an arc on $\{s, v\}$. Let w be the last internal vertex on this path.

First suppose that w is a bifurcation. Then we reverse the arc wv to vw using rNNI moves. To see that this is possible, note that w is a bifurcation and v a reticulation, and that there cannot be a nonelementary w-v path in N: this path would have to go via u and would form a directed cycle in combination with the s-w path in N. Hence, reversing wv to vw is an arc flip, which by Lemma 2 can be reproduced using rNNI moves. We can then apply a type-(1) rNNI move $(us, uv, vt \to ut, uv, vs)$ and obtain an acyclic network N'' with the same underlying unrooted network as N'. See Figure S4.

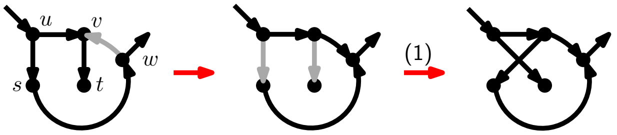

Fig S4. Avoiding directed cycles in Case (1) when w is a bifurcation. Note that the arc leaving w that does not point at v could point at t.

Now suppose that w is a reticulation. Let $(s = x_0, x_1, x_2, \ldots, x_k = w)$ be a longest s-w path. Let x_i be the first reticulation on this path. Note that there cannot be a nonelementary x_{i-1}-x_i path because otherwise there would be a longer s-w path. Hence, we can flip the orientation of arc $x_{i-1}x_i$ using rNNI moves by Lemma 2. We repeat this procedure until there is no s-w path. Then we apply type-(1) rNNI move $(us, uv, vt \to ut, uv, vs)$ and obtain an acyclic network N'' with the same underlying unrooted network as N'. See Figure S5.

Case (2). $(su, uv, tv \to sv, uv, tu)$ and there is a u-t path in N, see Figure S6.

First suppose that t is a bifurcation. Then we flip arc tv to vt using rNNI moves. To see that this is possible, assume that there were a nonelementary t-v path. This path

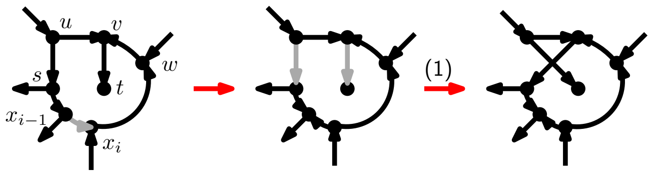

Fig S5. Avoiding directed cycles in Case (1) when w is a reticulation.

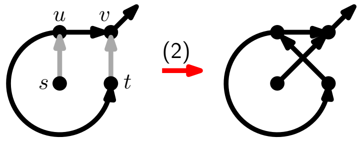

Fig S6. A cycle-creating rNNI move of type (2).

would then have to enter v through u. However, since there is also a u-t path, this would imply the existence of a directed cycle in N. Hence, we can perform an arc flip on tv via rNNI moves by Lemma 2. Then we can apply a type-(3*) rNNI move $(su, uv, vt \to sv, vu, ut)$. This move is possible because there can be no nonelementary u-v path since v has indegree 1. We have thus obtained an acyclic network N'' with the same underlying unrooted network as N'. See Figure S7.

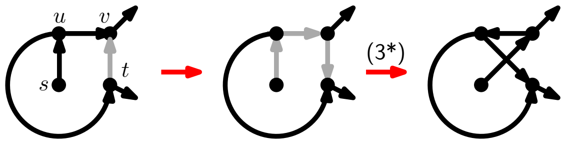

Fig S7. Avoiding directed cycles in Case (2) when t is a bifurcation.

Now suppose that t is a reticulation. Let $(u = x_0, x_1, x_2, \ldots, x_k = t)$ be a longest u-t path in N. Let x_i be the first reticulation on this path. Then we flip arc $x_{i-1}x_i$ (which is again possible since we chose a longest u-t path) using rNNI moves, and keep repeating this procedure until there are no u-t paths left. Then we apply type-(2) rNNI move $(su, uv, tv \to sv, uv, tu)$ and obtain an acyclic network N'' with the same underlying unrooted network as N'. See Figure S8.

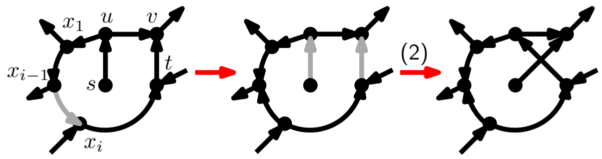

Fig S8. Avoiding directed cycles in Case (2) when t is a reticulation.

Case (3*). $(su, uv, vt \to sv, vu, ut)$ and there is a nonelementary u-v path in N, see Figure S9.

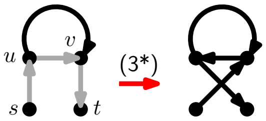

Fig S9. A cycle-creating rNNI move of type (3*).

First suppose there exists at least one nonelementary u-v path where the last internal vertex w of the path is a bifurcation. Then we flip arc wv using rNNI moves. This is possible by Lemma 2 because a nonelementary w-v path would have to pass

through u and hence imply the existence of a directed cycle in N involving u and w. After that, there can be no nonelementary u-v path since v has only one incoming arc which comes from u. Therefore, we can apply the type-(3^*) move ($su, uv, vt \to sv, vu, ut$) and we are done. See Figure S10.

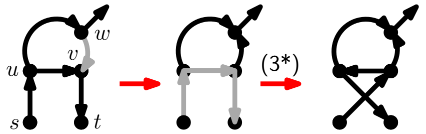

Fig S10. Avoiding directed cycles in Case (3) when w is a bifurcation.

Now suppose that in all nonelementary u-v paths the last internal vertex is a reticulation. Then we take a longest u-v path ($u = x_0, x_1, x_2, \dots, x_k = v$) and let x_i be the first reticulation on this path. Then we flip arc $x_{i-1}x_i$, which is again possible since we chose a longest u-v path. We repeat this procedure until there are no nonelementary u-v paths left. Then we can apply the type-(3^*) move ($su, uv, vt \to sv, vu, ut$) and we are done. See Figure S11.

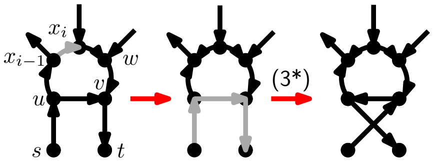

Fig S11. Avoiding directed cycles in Case (3) when w is a reticulation.

Case (4). ($us, uv, tv \to vs, uv, tu$) and there is an s-t path in N, see Figure S12.

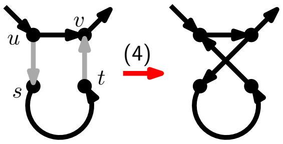

Fig S12. A cycle-creating rNNI move of type (4).

First suppose that t is a bifurcation. Then we flip arc tv using rNNI moves. As before, this is possible because a nonelementary t-v path would need to pass through u and hence imply the existence of a directed cycle in N. Then we apply a type-(1) rNNI move ($us, uv, vt \to ut, uv, vs$). This is possible because any s-v path would have to pass through u and hence imply the existence of a directed cycle in N. See Figure S13.

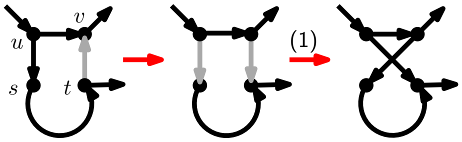

Fig S13. Avoiding directed cycles in Case (4) when t is a bifurcation.

Now suppose that t is a reticulation. Then we take a longest s-t path ($s = x_0, x_1, x_2, \dots, x_k = t$) and let x_i be the first reticulation on this path. If $i = 0$, i.e. if s is a reticulation, then we flip arc us, apply a type-(2) move ($su, uv, tv \to sv, uv, tu$) and we are done. Otherwise, we flip arc $x_{i-1}x_i$ which is, as before, possible since we chose a longest s-t path, and we repeat the procedure until there are no s-t paths left. Then we can apply the type-(4) move ($us, uv, tv \to vs, uv, tu$) and we are done. See Figure S14. □

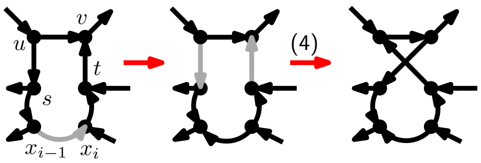

Fig S14. Avoiding directed cycles in Case (4) when t is a reticulation.

Lemma 5. *For any nonempty X and $r \geq 1$, there exists a flip-friendly binary rooted network on X with r reticulations.*

Proof. Any network with just one reticulation is level-1, and thus, by Lemma 4, also flip-friendly. In order to prove the lemma for $r \geq 2$, we proceed as follows: we introduce a special type of rooted binary networks, the *laddered networks*, and then we show that (1) there are laddered networks on X with any number of reticulations $r \geq 2$, and (2) laddered networks are flip-friendly.

A *rooted ladder* is a binary rooted network that can be obtained in the following manner (see Fig. S15, left): take a directed path $P = p_1 p_2 \ldots p_r$ and another directed path $Q = q_1 q_2 \ldots q_r$, both on r vertices, and add an arc from p_i to q_i for each $i \in \{1, \ldots, r\}$. Then add a vertex x with children p_1 and q_1, and a vertex y with parents p_r and q_r. Finally add a root ρ whose only child is x and a leaf l whose parent is y. Note that the reticulations in a rooted ladder are the vertices of the Q path, and the y vertex. Clearly, for each $r \geq 2$ there exists a rooted ladder with r reticulations.

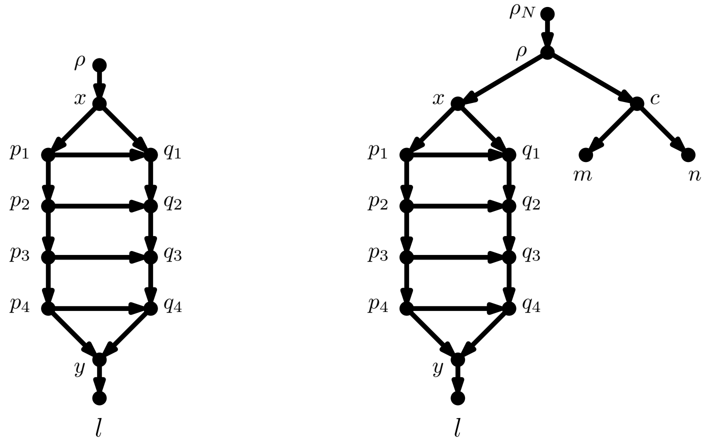

Fig S15. A rooted ladder L with 5 reticulations (left) and a laddered network (right), obtained by grafting L on the root arc of a rooted tree on $\{m, n\}$.

A *laddered network* is a rooted binary network obtained by taking a rooted binary tree N, then *grafting* a rooted ladder on the arc between the root of N and its child; if we denote these two nodes by ρ_N and c, respectively, and the root of the rooted ladder by ρ, this means replacing the arc $\rho_N c$ in N with two new arcs $\rho_N \rho$, ρc and then adding to N the remaining vertices and arcs of the rooted ladder. See Fig. S15 (right) for an example of laddered network. We can now prove the claims on laddered networks that are necessary to conclude our proof.

Claim (1). *For any nonempty X and $r \geq 2$, there exists a laddered network on X with r reticulations.*

Let l be any element of X, and L be the rooted ladder having r reticulations and l as its leaf. Then let N be a laddered network obtained by taking a binary rooted tree on $X \setminus \{l\}$, then grafting L on the branch between the root of this tree and its child. If

$X \setminus \{l\} = \emptyset$, then let N simply be L. Thus, N is a laddered network on X with r reticulations.

Claim (2). *Laddered networks are flip-friendly.*

Let N be a laddered network on X, and N' another binary rooted network on X with the same underlying unrooted network. We show that N can be transformed into N' by only using arc flips.

First observe that in the "tree part" of N' all arcs will be oriented in the same way as in N. If this were not true, N' would be rooted in a different degree-1 node than N, and the two networks would not be on the same set X. Therefore the only arcs appearing in N but not in N' will be in its "ladder part". So consider this part of N, and let $x, y, l, p_1, \ldots, p_r, q_1, \ldots, q_r$ be the vertices in N described in the definition of a rooted ladder (see Fig. S15). For notational convenience let $p_0 = q_0 = x$, and let $p_{r+1} = q_{r+1} = y$.

Consider the set $W_0 = \{x, p_1, \ldots, p_r, q_1, \ldots, q_r, y, l\}$. The only vertex in W_0 with a neighbor outside of W_0 is x, and every vertex in W_0 has indegree at least 1 (as W_0 does not contain the root of N). Therefore if N' contains the arc p_1x or q_1x, it holds that for every $w \in W_0$ there exists $z \in W_0$ such that zw is an arc in N'. But this implies that N' has a cycle contained in W_0, a contradiction. Thus N' can have neither of the arcs p_1x, q_1x, and so instead N' must have arcs xp_1, xq_1.

We have thus shown that N' contains the arcs p_0p_1, q_0q_1. We will now show by induction that for each $i \in \{1, \ldots, r\}$, N' contains the arcs p_ip_{i+1} and q_iq_{i+1}.

Consider the set $W_i = \{p_i, \ldots, p_r, q_i, \ldots, q_r, y, l\}$. The only vertices in W_i with a neighbor outside of W_i are p_i and q_i. If N' contains the arc $p_{i+1}p_i$ then, as N' contains the arc $p_{i-1}p_i$, N' must also contain the arc p_iq_i. But then we have that for every $w \in W_i$ there exists $z \in W_i$ such that zw is an arc in N'. This implies that N' has a cycle contained in W_i, a contradiction. Thus N' cannot contain the arc $p_{i+1}p_i$. By a symmetric argument N' cannot contain the arc $q_{i+1}q_i$. Thus we have that for any N' and every $i \in \{0, \ldots, r\}$, N' contains the arcs p_ip_{i+1} and q_iq_{i+1}.

It follows that the only arcs in N that may not be in N' are the p_iq_i for some $i \in \{1, \ldots, r\}$. For any such arc there is no nonelementary p_i-q_i path in N, p_i is a bifurcation and q_i is a reticulation. Therefore, we can perform an arc flip on each arc in N and not in N', meaning that N' can be obtained from N by a sequence of arc flips.

□

Theorem 4. *Let N and N' be binary rooted networks. Then, N can be turned into N' with one rNNI move if and only if N can be turned into N' with one rSPR$_1$ move.*

Proof. We first prove that every rSPR$_1$ move is an rNNI move (which implies the *if* part of the theorem). In order to do this, we consider four different cases for the position of the recipient arc $x'y'$ relative to the donor arcs xz, zy (see Fig. 7 in the main text). We refer to Lemma 1 for the definitions of the rNNI types $(1), (1^*), \ldots, (4)$.

(a) $y' = x$, that is the recipient arc enters x. In this case the rSPR$_1$ move coincides with the rNNI $(x'x, xz, zy \to x'z, zx, xy)$, which is an rNNI of type (3^*) with $s = x', u = x, v = z, t = y$.

(b) $x' = x$, that is the recipient arc exits x. In this case the rSPR$_1$ move coincides with the rNNI $(xy', xz, zy \to zy', xz, xy)$, which is an rNNI of type (1) with $s = y', u = x, v = z, t = y$.

(c) $x' = y$, that is the recipient arc exits y. In this case the rSPR$_1$ move coincides with the rNNI $(xz, zy, yy' \to xy, yz, zy')$, which is an rNNI of type (3^*) with

$s = x, u = z, v = y, t = y'$.

(d) $y' = y$, that is the recipient arc enters y. In this case the rSPR$_1$ move coincides with the rNNI $(xz, zy, x'y \to xy, zy, x'z)$, which is an rNNI of type (2) with $s = x, u = z, v = y, t = x'$.

We now proceed to prove the *only if* direction of the theorem. That is, if N can be turned into N' with one rNNI move, then the same can be done with one rSPR$_1$ move. Similarly to above, we consider each possible type of rNNI in turn.

(1) $(us, uv, vt \to ut, uv, vs)$. This rNNI move is an rSPR$_1$ with donor arcs uv, vt and recipient arc us.

(1*) $(us, uv, vt \to ut, vu, vs)$, where v is a reticulation in N. Let x be the only parent of u in N, and x' the parent of v other than u. Now consider the rSPR$_1$ move with donors xu, uv and recipient arc $x'v$, that is $[xu, uv, x'v \to x'u, uv, xv]$. The resulting network is the same as N' (formally, isomorphic to N'), the network produced by the rNNI above: both networks contain the arcs $x'\alpha, x\beta, \alpha\beta, \alpha s, \beta t$, with $\alpha = v, \beta = u$ in N', and $\alpha = u, \beta = v$ in the network produced by the rSPR$_1$.

(2) $(su, uv, tv \to sv, uv, tu)$. This rNNI move is an rSPR$_1$ with donor arcs su, uv and recipient arc tv.

(2*) $(su, uv, tv \to sv, vu, tu)$, where u is a bifurcation in N. Let y be the only child of v in N, and y' the child of u other than v. Now consider the rSPR$_1$ move with donors uv, vy and recipient arc uy', that is $[uv, vy, uy' \to uv, vy', uy]$. The resulting network is the same as N' (formally, isomorphic to N'), the network produced by the rNNI above: both networks contain the arcs $s\alpha, t\beta, \alpha\beta, \alpha y, \beta y'$, with $\alpha = v, \beta = u$ in N', and $\alpha = u, \beta = v$ in the network produced by the rSPR$_1$.

(3) $(su, uv, vt \to sv, uv, ut)$, where u is a reticulation and v a bifurcation in N. Let x' be the parent of u other than s in N, and y the child of v other than t. Now consider the rSPR$_1$ move with donors uv, vy and recipient arc $x'u$, that is $[uv, vy, x'u \to x'v, vu, uy]$. The resulting network is the same as N' (formally, isomorphic to N'), the network produced by the rNNI above: both networks contain the arcs $x'\alpha, s\beta, \alpha\beta, \alpha t, \beta y$, with $\alpha = u, \beta = v$ in N', and $\alpha = v, \beta = u$ in the network produced by the rSPR$_1$.

(3*) $(su, uv, vt \to sv, vu, ut)$. This rNNI move is an rSPR$_1$ with donor arcs su, uv and recipient arc vt. Interestingly, it is also an rSPR$_1$ with donor arcs uv, vt and recipient arc su.

(4) $(us, uv, tv \to vs, uv, tu)$. Let y be the only child of v in N, and x' the only parent of u. Now consider the rSPR$_1$ move with donors uv, vy and recipient arc $x'u$, that is $[uv, vy, x'u \to x'v, vu, uy]$. The resulting network is the same as N' (formally, isomorphic to N'), the network produced by the rNNI above: both networks contain the arcs $x'\alpha, t\alpha, \alpha\beta, \beta s, \beta y$, with $\alpha = u, \beta = v$ in N', and $\alpha = v, \beta = u$ in the network produced by the rSPR$_1$.

□

Proposition 2. *Let N be a binary rooted network. Within N, let e_{BB} denote the number of arcs from a bifurcation to a bifurcation, e_{BR} the number of arcs from a bifurcation to a reticulation, e_{RB} the number of arcs from a reticulation to a bifurcation, and e_{RR} the number of arcs from a reticulation to a reticulation. Then, the number of different binary rooted networks that can be obtained from N by one rNNI move is at most $2(e_{BB} + e_{RR}) + 3e_{BR} + 4e_{RB}$.*

Proof. Every rNNI move applied to N must be around some arc uv in N, where both u and v are internal vertices (that is, neither the root or a leaf). Thus, u and v are either bifurcations or reticulations. To prove the statement, we consider the four possible assignments of u and v to these categories.

In the following, we show that if u and v are both bifurcations (case BB) or both reticulations (case RR), then at most 2 networks can be obtained with an rNNI move around uv (top two lines in Fig. 8 in the main text). If instead u is a bifurcation and v a reticulation (case BR), then at most 3 networks can be obtained (third line in Fig. 8). Finally, if u is a reticulation and v a bifurcation (case RB), then at most 4 networks can be reached with an rNNI move around uv (bottom line in Fig. 8). These observations allow us to obtain the upper bound of $2(e_{BB} + e_{RR}) + 3e_{BR} + 4e_{RB}$ on the size of the rNNI neighborhood. In the following four paragraphs, we provide the detailed (but tedious) proofs for cases BB, RR, BR and RB.

Case BB. If both u and v are bifurcations, name the vertices adjacent to u or v, and networks N_1 and N_2 in the way described in Fig. 8 (top line), where we may have $\beta = \gamma$ or $\beta = \delta$, but no other equality between vertices (any such equality would either imply a cycle or parallel arcs). The only rNNI moves that can be applied to N are of type (1) and (3*), as all other rNNI types require that either u or v is a reticulation. The type-(1) move $(u\beta, uv, v\gamma \to u\gamma, uv, v\beta)$ and the type-(3*) move $(\alpha u, uv, v\delta \to \alpha v, vu, u\delta)$ result in network N_1, whereas the type-(1) move $(u\beta, uv, v\delta \to u\delta, uv, v\beta)$ and the type-(3*) move $(\alpha u, uv, v\gamma \to \alpha v, vu, u\gamma)$ result in network N_2. Note that if $\beta = \gamma$ or $\beta = \delta$, then some of the moves above may not be applicable. Thus at most 2 networks can be obtained with an rNNI move around uv in this case.

Case RR. If both u and v are reticulations, name the vertices adjacent to u or v, and networks N_1 and N_2 in the way described in Fig. 8 (2nd line), where we may have $\beta = \gamma$ or $\alpha = \gamma$, but no other equality between vertices. The only rNNI moves that can be applied to N are of type (2) and (3*), as all other rNNI types require that either u or v is a bifurcation. The type-(2) move $(\beta u, uv, \gamma v \to \gamma u, uv, \beta v)$ and the type-(3*) move $(\alpha u, uv, v\delta \to \alpha v, vu, u\delta)$ result in network N_1, whereas the type-(2) move $(\alpha u, uv, \gamma v \to \gamma u, uv, \alpha v)$ and the type-(3*) move $(\beta u, uv, v\delta \to \beta v, vu, u\delta)$ result in network N_2. Note that if $\beta = \gamma$ or $\alpha = \gamma$, then some of the moves above may not be applicable. Thus at most 2 networks can be obtained with an rNNI move around uv in this case.

Case BR. If u is a bifurcation and v a reticulation, name the vertices adjacent to u or v, and networks N_1, N_2 and N_3 in the way described in Fig. 8 (3rd line), where we may have $\alpha = \beta$, $\delta = \gamma$, $\gamma = \beta$. Here type-(3) rNNI moves cannot be applied, as they require that u is a reticulation. All other moves, if applicable, result in one among N_1, N_2 and N_3: the only type-(1) move $(u\gamma, uv, v\delta \to u\delta, uv, v\gamma)$ results in N_3; the only type-(1*) move $(u\gamma, uv, v\delta \to u\delta, vu, v\gamma)$ results in N_2; the only type-(2) move $(\alpha u, uv, \beta v \to \beta u, uv, \alpha v)$ results in N_2; the only type-(2*) move $(\alpha u, uv, \beta v \to \beta u, vu, \alpha v)$ results in N_3; the only type-(3*) move $(\alpha u, uv, v\delta \to \alpha v, vu, u\delta)$ results in N_1; the only type-(4) move $(u\gamma, uv, \beta v \to v\gamma, uv, \beta u)$ results in N_1. Once again (when some of the involved vertices are not distinct, or when N contains a γ-β path) some of the moves above may not be applicable. Thus at most 3 networks can be obtained with an rNNI move around uv in this case.

Case RB. If u is a reticulation and v a bifurcation, name the vertices adjacent to u or v in the way described in Fig. 8 (bottom line). The only rNNI moves that can be applied to N are of type (3) and (3*), as all other rNNI types require that either u is a bifurcation or v a reticulation. They result in one among N_1, N_2, N_3 and N_4: the type-(3) move $(\beta u, uv, v\gamma \to u\gamma, uv, \beta v)$ and the type-(3*) move

$(\alpha u, uv, v\delta \to \alpha v, vu, u\delta)$ result in N_1; the type-(3) move $(\beta u, uv, v\delta \to u\delta, uv, \beta v)$ and the type-(3*) move $(\alpha u, uv, v\gamma \to \alpha v, vu, u\gamma)$ result in N_2; the type-(3) move $(\alpha u, uv, v\gamma \to u\gamma, uv, \alpha v)$ and the type-(3*) move $(\beta u, uv, v\delta \to \beta v, vu, u\delta)$ result in N_3; the type-(3) move $(\alpha u, uv, v\delta \to u\delta, uv, \alpha v)$ and the type-(3*) move $(\beta u, uv, v\gamma \to \beta v, vu, u\gamma)$ result in N_4. Note that in this case, no equality between any of the named vertices can hold. Moreover, because no nonelementary u-v path can exist in N, none of the moves above can create a cycle. Thus all of the moves above are applicable, and exactly 4 networks can be obtained with an rNNI move around uv in this case. □

About the size of rNNI neighborhoods.

We now give a family of networks N^k, illustrated in Fig. S16, whose rNNI neighborhood has size logarithmic in the number of arcs, in contrast with the upper bound given in the Results section, which is linear in the number of arcs.

Each network N^k is built by taking two copies T_1^k and T_2^k of a complete binary rooted tree with 2^k leaves. For each pair of leaves u and v, add all possible arcs from the copies of u and v in T_1^k to the copies of u and v in T_2^k. Finally, replace each arc uv in T_k^2 with the arc vu, so that the resulting network is binary with a single root and a single leaf. This completes the construction of N^k (see Fig. S16).

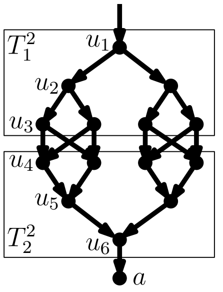

Fig S16. The network N^2, illustrating a family of networks N^k with $O(2^k)$ arcs and only $O(k)$ networks in the rNNI neighborhood.

For all $k > 0$, as the number of arcs of a complete binary subtree with 2^k leaves is $2^{k+1} - 1$, the number of arcs of N^k is $2 \times (2^{k+1} - 1) + 2 \times 2^k$, that is $3 \times 2^{k+1} - 2$. However, because of the extreme symmetry of this network, all arcs that lie at the same height in the network are effectively indistinguishable, implying that rNNI moves around different arcs often result in the same network. More precisely, consider two arcs uv and $u'v'$ whose sources u and u' are at the same distance d from the root, for $d \in \{1, \dots, 2k+1\}$. It is easy to see that the set of networks that can be obtained by one rNNI move around uv is the same as the set obtained by one rNNI move around $u'v'$, or around any other arc whose source is at distance d of the root. Thus, as Prop. 2 implies that there are at most 4 networks in each of these sets, the size of the rNNI neighborhood of N^k is at most $4(2k+1)$. This proves that the size of the rNNI neighborhood of N^k is logarithmic in its number of arcs.

Finally, in Fig. S17, we illustrate the rNNI neighborhood of the network N_3 of Fig. 9 in the main text, which consists of 12 networks. Now note that because $e_{BB} = 3, e_{BR} = 2$, Prop. 2 gives an upper bound of exactly 12, showing that this bound is tight in this case.

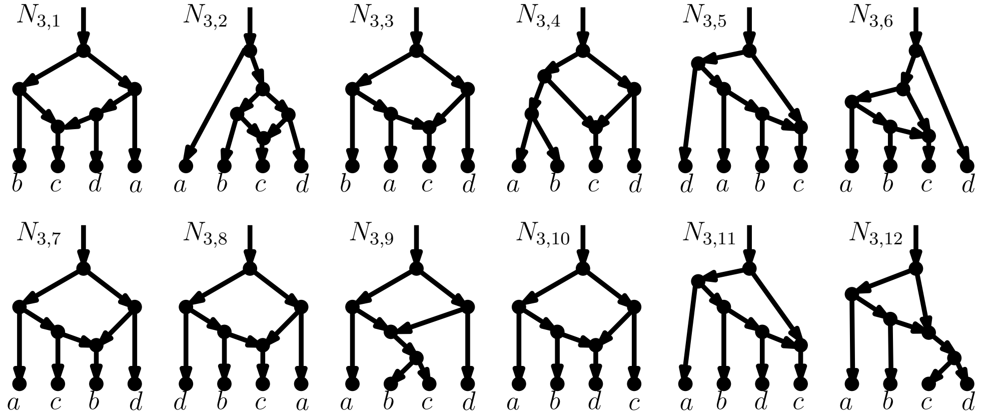

Fig S17. The rNNI neighborhood of network N_3 of Fig. 9 in the main text: $N_{3,1}$ and $N_{3,2}$ are obtained by rNNI moves of type BB around arc vv_1 of N_3, $N_{3,3}$ and $N_{3,4}$ are obtained by rNNI moves of type BB around arc v_1v_2, $N_{3,5}$ and $N_{3,6}$ are obtained by rNNI moves of type BB around arc vv_4, $N_{3,7}$, $N_{3,8}$ and $N_{3,9}$ are obtained by rNNI moves of type BR around arc v_2v_3 and $N_{3,10}$, $N_{3,11}$ and $N_{3,12}$ are obtained by rNNI moves of type BR around arc v_4v_3.
